# Diagnosis, treatment, and management of pericardial effusion- review

**DOI:** 10.1016/j.amsu.2022.104142

**Published:** 2022-07-09

**Authors:** Naser Yamani, Ayesha Abbasi, Talal Almas, Farouk Mookadam, Samuel Unzek

**Affiliations:** a Department of Medicine, John H Stroger Jr. Hospital of Cook County, Chicago, IL, 60612, USA; b Department of Medicine, RCSI University of Medicine and Health Sciences, Dublin, Ireland; c Department of Cardiovascular Medicine, Banner University Medical Center, Phoenix, AZ, USA

**Keywords:** Pericardial effusion, Pericardiocentesis, Pericardial window, Sclerosing therapy

## Abstract

The hemodynamic stability of the heart and pericardium are maintained by the pericardial fluid of volume ∼10–50 ml. Pericardial effusion is associated with the abnormal accumulation of pericardial fluid in the pericardial cavity. Numerous imaging techniques are utilized to evaluate pericardial effusion including chest X-ray, electrocardiogram, transthoracic echocardiography, computed tomography scan, cardiac magnetic resonance imaging, and pericardiocentesis. Once diagnosed, there are numerous treatment options available for the management of patients with pericardial effusion. These include various invasive and non-invasive strategies such as pericardiocentesis, pericardial window, and sclerosing therapies. In recent times, few studies have been conducted to evaluate the safety and efficacy of each approach in routine clinical practice. In this review, we review the role of different modalities in the diagnosis of pericardial effusion while highlighting existing therapies aimed at the management and treatment of pericardial effusion.

## Highlights

• Numerous imaging techniques are utilized to evaluate pericardial effusion (PE) including chest X-ray, electrocardiogram, transthoracic echocardiography, CT scan, cardiac MRI, and pericardiocentesis.
• Multiple treatment options are available for the management of patients with PE including pericardiocentesis, pericardial window, and sclerosing therapies.
• Recent studies have evaluated the safety and efficacy of various diagnostic and management techniques in routine clinical practice.
• Further research is needed to investigate the optimal diagnostic and treatment options for patients with PE.

## 1 Introduction

Pericardial effusion refers to the abnormal accumulation of pericardial fluid in the pericardial cavity [1,2]. Under physiologic conditions, the pericardial space contains 10–50 ml of serous pericardial fluid, which provides lubrication, thereby reducing friction on the movement of cardiac chambers [1]. Pericardial effusion represents a relatively common finding in everyday clinical practice which may present by chance or as a life-threatening emergency. In accordance with the varying clinical presentation of pericardial effusion, numerous causes are identified, which are broadly classified as inflammatory and non-inflammatory. Additionally, they may be attributable to idiopathic pericarditis and infections, trauma, radiation, neoplasms, autoimmune diseases, cardiac injury, and noxious substances [[2], [3], [4]]. In severe cases, cardiac tamponade may develop with the progression of pericardial effusion resulting in circulatory collapse.

In patients with a differential diagnosis of pericardial effusion, the echocardiogram is the most widely available and reliable technique to authenticate the presence and severity of pericardial effusion [5,6]. Chest X-ray, electrocardiogram (ECG), computed tomography (CT), or cardiac magnetic resonance imaging (MRI) are alternate imaging modalities that aid in the diagnosis of pericardial effusion. Increased understanding of the pathophysiology of pericardial effusion has led to the development of therapies targeting the underlying cause. Cardiologists have frequently debated the ideal technique to manage pericardial effusion which ranges from sclerosing therapies to pericardiocentesis and pericardial window. Few studies [[7], [8], [9], [10], [11], [12]] have attempted to compare different approaches and their feasibility in regular clinical practice (Table 1).

**Table 1:** Characteristics of studies reviewing and comparing the efficacy of different percutaneous techniques to resolve pericardial effusion.

Study Title	Publication Year	Intervention	Primary Endpoint	Age (years)	Hypertension (%)	Diabetes (%)	Patient Population
Langdon et al. [7]	2016	Subxiphoid (n = 127) vs. Thoracotomy (n = 52)	Time to extubation (hours)	73.6 ± 11.6 vs 72.3 ± 12.8	79 (62.2%) vs. 37 (71.2%)	23 (18.1%) vs. 13 (25.0%)	patients who underwent a pericardial window operation using either a subxiphoid or left anterior thoracotomy incision.
Balla et al. [8]	2020	Subxiphoid (n = 31) vs. Transpleural (n = 15)	operative outcomes in patients	53.3 (43.1–58.4) vs. 41.5 (32.7–49.8)^a^	8 (25.8%) vs. 5 (33.3%)	5 (16%) vs. 3 (20%)	patients who underwent a pericardial window excluding those who underwent recent cardiothoracic surgery or trauma.
Nguyen et al. [10]	2011	subxiphoid pericardial window (n = 60)	survival rates	60	N/A	N/A	patients who underwent a surgical pericardial window for pericardial effusion.
Celik et al. [9]	2012	pericardial window formation via mini-thoracotomy (n = 53)	Risk factors affecting survival	55.2 ± 12.97	N/A	N/A	cancer patients with pericardial tamponade treated by pericardial window formation
Tsang et al. [11]	2002	Consecutive echo-guided pericardiocenteses (n = 977)	Procedural success (period 1 vs 2 vs 3)	49 ± 14 vs. 52 ± 13 vs. 57 ± 14	N/A	N/A	Patients from the Mayo Clinic Echocardiographic-guided Pericardiocentesis Registry who underwent therapeutic echo-guided pericardiocenteses for treatment of clinically significant pericardial effusions
Piehler et al. [12]	1985	Pericardial resection (n = 145)	relationship between the extent of resection and the development of late comptications	50.5	N/A	N/A	patients who underwent operation for effusive pericardial disease

a Indicates Median (IQR).

Accordingly, this review has two primary aims: First, we review the role of different techniques in the diagnosis of pericardial effusion. Second, we review existing therapies aimed at the management and treatment of pericardial effusion with a wide spectrum of underlying etiologies.

## 2 Fundamentals of pericardial effusion

**Fig. 1:**
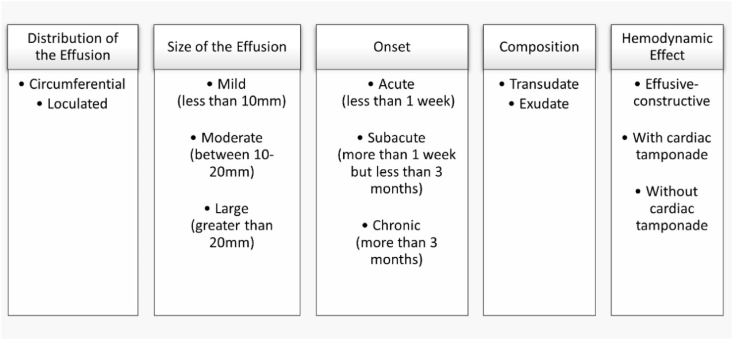
Principle classifications of PE.

The hemodynamic stability of the heart and pericardium are maintained by the pericardial fluid of volume ∼10–50 ml. The fluid acts as a lubricant between the two layers of the pericardium, permitting frictionless movement of the cardiac chambers without interrupting the activity and position of the surrounding structures in the mediastinum. Disrupted drainage of the pericardial fluid (transudate) or excessive production (exudate), due to inflammatory or non-inflammatory mechanisms leads to accumulation of fluid in the pericardial sac. Fluid accumulation of >50 mL is classified as a pericardial effusion which manifests as a compromised hemodynamic condition of the patient. A wide variety of virulent and bacterial infections, cardiovascular injuries, malignancies, trauma, hypothyroidism, renal failure, and other underlying comorbidities or idiopathic conditions contribute to the accumulation. Therefore, pericardial effusion can be classified according to five principal features (Fig. 1).

## 3 Diagnosis of pericardial effusion

Numerous imaging techniques are utilized to evaluate pericardial effusion. The primary evaluation of a pericardial effusion should focus on the assessment of the size of effusion, progression to cardiac tamponade, presence of coexisting pericarditis, duration of effusion, and presence of underlying diseases that may be responsible for effusion e.g., cancer, tuberculosis, inflammatory diseases, and metabolic disorders [1,13]. Since many patients present with chest pain and dyspnea, chest X-rays are usually obtained in clinical settings. The chest radiograph may fail to directly recognize a pericardial effusion. If there is a large effusion, the heart may appear boot-shaped also known as the “water bottle sign”. However, it is non-specific with a supportive role and low sensitivity [14].

The electrocardiogram (ECG) findings can also aid in establishing a differential diagnosis of pericardial effusion. Small effusions may manifest as non-specific ST-segment changes while large effusions or cardiac tamponade may clinically present as electrical alternans which is a non-sensitive but specific indication. This finding refers to beat-to-beat alterations in QRS complexes attributable to the movement of the heart in the pericardial fluid [15]. In addition, PR depressions or diffuse ST elevations may be observed in pericarditis-related pericardial effusion [5,16].

For decades, transthoracic echocardiography is the diagnostic modality of choice for the definitive evaluation of pericardial effusion (Class I recommendation, LOE C according to 2015 ESC guidelines) [2]. Echocardiography provides a detailed evaluation of the size and location of the effusion while assessing the progression to cardiac tamponade [14,17]. Pericardial effusion presents as an anechoic fluid located between the pericardium and epicardium. On the contrary, effusions with a clot or exudate may show a varied appearance [18,19]. These findings are differentiated from epicardial fat which shifts with the myocardium during the cardiac cycle and is more hyperechoic [19]. If a pericardial effusion is seen only during systole, it is classified as physiologic. Effusion size can be obtained through echocardiography by estimating the cardiac proportions at end-diastole. Weitzman et al. [20] classified effusions by size as follows: <10 mm small, 10–20 mm moderate, 20–25 mm large, and >25 mm very large [20]. Furthermore, echocardiography provides an assessment of cardiac tamponade by identifying conditions where intra-cardiac pressures are lesser than intra-pericardial pressures. Accordingly, right atrial free-wall collapse or inversion during systole, right ventricular free wall collapse in diastole, raised septal bowling into right ventricle during exhalation and left ventricle during inhalation, and higher flow across the mitral valve during exhalation and tricuspid valve during inhalation indicates the presence of pericardial tamponade [5].

Malignant pericardial effusion is a serious complication of malignant tumors with a 10–21% incidence upon autopsy [21,22]. Therefore, oncologic patients are advised to undergo continuous echocardiographic monitoring before, during, and after the completion of therapy. In addition, due to the long-lasting deleterious effects of radiation therapy, continual echocardiographic evaluation is advised [23].

Although echocardiography is the diagnostic imaging modality of choice for the assessment of pericardial effusion, a CT scan can be utilized when more accurate details about the location, extent, and quantity of pericardial fluid are required, or when the effusion is complex or has clots [19,[24], [25], [26]]. First, a CT scan enables the identification of a possible thoracic neoplasm and epicardial fat. Second, it draws a clear distinction between pericardial thickening and pericardial effusion. In addition, a CT scan accurately defines regions that are challenging to discern with echocardiography. Lastly, CT scans can differentiate pericardial effusion from conditions with similar presentations on routine imaging e.g., pleural effusions, lower lobe atelectasis, and mediastinal abnormalities [3]. CT scans can also help delineate the composition of a pericardial effusion using the degree of CT attenuation of the pericardial fluid (Hounsfield units, HU) [27]. Attenuation values similar to water i.e., less than 10 HU are indicative of transudative effusions whereas higher attenuation values i.e., >60 HU are seen in hemorrhagic effusions. Attenuation values in the range of 10–60 HU are due to exudative effusions [28]. However, non-gated CT exams do not examine hemodynamic relations and may exaggerate cardiac dimensions [29].

Like CT, cardiac MRI also identifies clots or complex loculated effusions. It possesses the unique ability to classify pericardial effusion and associated masses [30]. In addition, it provides details of the anatomical and hemodynamic relations of the pericardium while assessing any concomitant inflammation of both the pericardium and myocardium [31]. Only limited patients should be referred for cardiac MRI e.g., patients with acoustic windows, indefinite echocardiographic results for constrictive disease, or those with persistent pericardial inflammation [19].

Pericardiocentesis is not essential for the diagnosis of the underlying cause of pericardial effusion. Indications for this diagnostic procedure include cardiac tamponade or effusion with suspected bacterial or neoplastic etiology (class I recommendation LOE C) [2]. Routine diagnostic analyses performed on the pericardial fluid include investigation of general chemistry, cytology, biomarkers (tumor markers, adenosine deaminase, and IFN-gamma), polymerase chain reaction for specific infectious agents, and microbiology assessment (acid-fast bacilli staining, mycobacterium cultures, aerobic and anaerobic cultures) [13]. Additional diagnostic testing is required when the underlying etiology is not evident. The possible procedures include skin testing for tuberculosis, screening for neoplasms, autoimmune diseases, thyroid disease, other metabolic disorders, and laboratory analysis for other infections. Pericardiocentesis is not indispensable and does not provide a specific diagnosis except for bacterial and metastatic etiologies [3].

Furthermore, in hemodynamically compromised patients, bedside transthoracic echocardiography (B-TTE) plays a cardinal role in providing immediate and crucial information regarding the progression of pericardial effusion to cardiac tamponade. B-TTE ameliorates the risk of delayed diagnosis thereby reducing mortality and morbidity rates in patients with a pericardial effusion. B-TTE allows the effective and timely identification of cardiac abnormalities in emergency situations such as thickened left-ventricular wall, small pericardial effusion, dysfunction of the valvular structures, and cardiac chamber dilations. However, a pilot study conducted in China by Lu et al. [32] assessed the diagnostic accuracy of on-site interpretation of these echocardiograms compared with tele-echocardiography by expert consultants. The results show that the advanced image analysis software and unperturbed working conditions facilitate accurate interpretation bridging of the gap created by the absence of radiologists in the emergency department.

## 4 Clinical approach to treatment

**Fig. 2:**
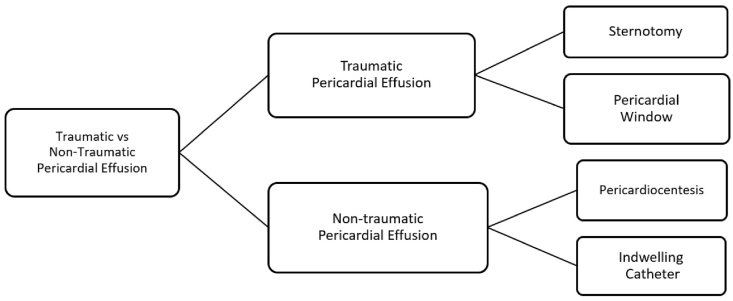
Triage for the management of pericardial effusion based on etiology.

Pericardial effusion is primarily managed by various invasive and non-invasive strategies. When pericardial fluid builds up, the chambers of the heart are unable to pump adequate stroke volume of blood, leading to pericardial effusion and tamponade, which is considered a cardiac emergency. The accumulated fluid can be removed through pericardiocentesis, in which a needle and a small catheter drain the excess fluid from the pericardial sac. The selection of procedures for the removal of excess fluid is determined by the etiology and size of the effusion (Fig. 2). Acute idiopathic pericardial effusion or pericardial effusion followed by viral pericarditis can be treated through a simple pericardiocentesis as it usually resolves within a few weeks with fewer chances of a tamponade occurring [33]. The latest 2015 ESC Guidelines on the management of pericardial diseases present a Class I Recommendation for performing pericardiocentesis for moderate to large effusions [2]. Under local anesthesia, pericardiocentesis can be performed through fluoroscopy or echocardiography guidance. The echocardiography-guided technique is widely used owing to its expeditious bedside accessibility and safety. In a study of 1000 patients, this approach showed a success rate of ∼97% [34]. Therefore, often in emergency settings such as a patient arriving with severe cardiac tamponade the first-line treatment is pericardiocentesis. Sometimes, after initial relief is obtained by pericardiocentesis, cases of large effusions or cardiac tamponade require prolonged drainage with an indwelling catheter, which may be continued for several days ahead. The removal of the catheter only takes place once the drainage becomes less than 20–30ml/24hr. In order to remove the loculated effusion, echo-guided pericardiocentesis with simultaneous fluoroscopy aids the procedure with safety through the halo phenomenon. This phenomenon allows a precise demarcation of the heart shadow, providing a safer approach while performing an echo-guided pericardiocentesis [35,36]. Tsang et al. [34] provide crucial data on pericardiocentesis-related complications such as ventricular tachycardia, injury to intercostal vessels, chamber laceration, and pneumothorax and mortality rate. In this study, the procedure when assisted by echocardiography, led to complications in 1.2% of patients, and related mortality was reported in 0.09% of the patients. However, pericardiocentesis is often associated with a high risk of recurrences, thus many clinical settings may use it as first-line therapy only for diagnostic purposes, but later stages of the illness are treated through other low-risk surgical procedures such as pericardial window, pericardial catheter drainage or sclerosing therapies [37].

While pericardiocentesis is usually the first-line therapy to drain excess fluid, in cases that make pericardiocentesis high risk e.g., in patients with malignant and recurrent pericardial effusion, a pericardial window is a safer alternative [19]. A pericardial window is a surgical technique usually preferred as an effective approach to avoid recurrent effusion in long term as compared to pericardiocentesis. This procedure involves the excision of pericardium allowing the accumulated fluid to drain directly into either the mediastinum known as subxiphoid drainage or into the thoracic cavity known as *trans*-pleural drainage. This window can be created either by conventional heart surgery or video-assisted thoracoscopy. Of the two, the subxiphoid approach is the preferred technique owing to lower postoperative pain, however, higher chances of recurrences exist with the selection of this procedure [7,[38], [39], [40]]. Balla et al. [8] extensively compare the overall safety and efficacy of the two pericardial window techniques. In this cohort of 46 patients, subxiphoid pericardial windows (n = 31) were performed more frequently than *trans*-pleural pericardial windows (n = 15). Although subxiphoid is the preferred technique among cardiothoracic surgeons, this study shows no significant association between mid-term recurrence, operative mortality, operative morbidity, pain medication requirements, and the two surgical techniques. The subxiphoid window, however, does provide an uncomplicated procedural plan as it can be performed in the simplest positioning and under local anesthesia. In the oncologic patient population, the likelihood of recurrent pericardial effusion is mostly due to the size and intensity of the effusion, and the type of associated cancer [8]. Recent studies highlight a similar approach toward the efficacy of the subxiphoid pericardial window. Langdon et al. [7] investigated patients who underwent subxiphoid pericardial window and found that the post-operative pain requirements were lower compared with patients who underwent *trans*-pleural drainage. Nguyen et al. [10] present a 1-year survival rate in 17% of the patients who underwent subxiphoid pericardial window creation, while other studies show survival rates of ∼23%–45% [9,12,41]. Hence, the common selection of subxiphoid pericardial window may not be due to institutional or surgeon preference. Data provided across recently conducted studies support the selection of this subset owing to a lower incidence of pericardial effusion recurrence and mortality.

In clinical practice, while numerous percutaneous techniques are commonly used as the first line of therapy to treat pericardial effusion, cardiologists are also inclined towards pharmaceutical therapies for critical cases, specifically cases of recurrence in malignant pericardial effusion. Sclerosing therapy use agents such as colchicine, bleomycin, doxycycline, tetracycline, cisplatin, etc. to induce inflammatory adhesion of the pericardial layers preventing re-accumulation of the pericardial fluid. The early and effective treatment of malignant pericardial effusion is possible through sclerosing therapy as it mostly remains undiagnosed until cardiac tamponade compromises the hemodynamic stability of the patient. Administration of sclerosing agents, especially tetracycline has contributed to palliation of symptoms along with increasing survival rate [42,43]. Another widely used sclerosing agent, bleomycin which also acts as a chemotherapeutic agent, has been compared to tetracycline in human clinical trials [44,45]. Results from these clinical trials show contradictory data, where Ruckdeschel et al. [44] present a higher recurrence rate associated with the use of tetracycline compared with bleomycin, whereas Walker-Renard et al. [45] suggested that tetracycline is superior to bleomycin. On the contrary, Vatikas et al. [40] and Yano et al. [46] reported a 100% success rate of bleomycin in 15 patients for controlling the malignant pericardial effusion across various types of primary cancer. In addition to these benefits, an on-set of effusive-constrictive pericarditis has been observed after sclerosing therapy was chosen to treat malignant pericardial effusion [47]. Hence, sclerosing therapy should be avoided in patients who have a relatively good life expectancy. The contradictory results warrant future pragmatic clinical trials and meta-analyses to assess the efficacy of different sclerosing agents.

## 5 Prognosis

The prognosis of pericardial effusion is determined by the underlying etiology [33,[48], [49], [50]]. Therefore, therapies should be targeted at specific causative factors. Very poor prognosis with a high mortality rate is seen in fungal or bacterial pericarditis while idiopathic/viral pericarditis shows a good prognosis. Patients with neoplastic pericardial effusion show a particularly poor prognosis due to advanced disease. In patients with pericardial effusion secondary to connective tissue diseases, the prognosis depends on the severity of those diseases. Lastly, chronic idiopathic pericardial effusion has a good prognosis but with an associated risk of tamponade [33].

## 6 Conclusion

Pericardial effusion is a common problem faced by physicians in everyday clinical settings. It has numerous underlying complex etiologies and clinical presentation varies from asymptomatic to severe cardiac tamponade. Echocardiography remains the imaging modality of choice for the diagnosis of pericardial effusion. Once diagnosed, there are numerous treatment options available for the management of patients with pericardial effusion. Several studies concur with the use of pericardiocentesis as the first line of therapy across all etiologies. However, at present, further research is required to establish the alternative use of sclerosing agents for the treatment of malignant pericardial effusion. Future, updated, and novel guidelines will provide further insight to help guide future clinical use.

## Ethical approval

N/A.

## Sources of funding

None to declare.

## Author contributions

Naser Yamani and Samuel Unzek – Conceptualization and designing the study.

Ayesha Abbasi and Talal Almas-drafting of the manuscript and data collection.

Naser Yamani and Farouk Mookadam – final review and approval for submission.

## Registration of research studies

1. Name of the registry:

2. Unique Identifying number or registration ID:

3. Hyperlink to your specific registration (must be publicly accessible and will be checked):

## Guarantor

Talal Almas.

Talalalmas.almas@gmail.com.

## Consent

N/A.

## Declaration of competing interest

None to declare.
